# Case report: diaphragmatic hernia after epicardial catheter ablation

**DOI:** 10.1093/ehjcr/ytag439

**Published:** 2026-06-09

**Authors:** Ole-Gunnar Anfinsen, Trine Synnøve Fink, Alexander Øye, Rasmus Broby Johansen, Magnus Hølmo Fasting

**Affiliations:** Department of Cardiology, Arrhythmia Section, Heart and Lung Clinic, Oslo University Hospital Rikshospitalet, Box 4950 Nydalen, Oslo N-0424, Norway; Department of Cardiology, Arrhythmia Section, Heart and Lung Clinic, Oslo University Hospital Rikshospitalet, Box 4950 Nydalen, Oslo N-0424, Norway; Department of Radiology, Vestre Viken Hospital Trust, Box 800, N-3004 Drammen, Norway; Division of Emergencies and Critical Care, Department of Anesthesiology and Intensive Care, Oslo University Hospital, Ullevål, Box 4950 Nydalen, Oslo N-0424, Norway; Department of Pediatric and Gastrointestinal Surgery, Oslo University Hospital, Ullevål, Box 4950 Nydalen, Oslo N-0424, Norway

**Keywords:** Case report, Catheter ablation, Ventricular tachycardia, Epicardial access, Procedural complication, CT thoracic angiography

## Abstract

**Background:**

Catheter ablation from the epicardial space is increasingly utilized to treat ventricular tachycardias (VTs). We report a rare complication.

**Case summary:**

A 67-year-old male with dilated cardiomyopathy and frequent, exercise-induced VTs had been ablated once with endocardial and twice with epicardial access. Six weeks after the last procedure, he was hospitalized with abdominal and back pain due to an iatrogenic diaphragmatic hernia with displacement of the stomach and spleen into the left thoracic cavity. Following an acute exacerbation with cardiac arrest and splenic bleeding, he underwent successful surgery with splenectomy, reposition of the stomach, and direct suture of the diaphragmatic hernia.

**Discussion:**

This is a rare complication, but notable for its delayed clinical manifestation several weeks after the procedure and necessitates urgent surgical treatment.

Learning points

- Epicardial catheter ablation may rarely cause collateral diaphragmatic injury.
- Iatrogenic diaphragmatic rupture may occur several weeks after an epicardial catheter ablation procedure and requires urgent surgical therapy.

## Introduction

Catheter ablation is increasingly used to treat ventricular tachycardias (VTs) in patients with structural heart disease.^1^ Endocardial ablation is performed transvenously from the groin with transseptal access and/or retrogradely through the aorta. However, particularly for patients with non-ischaemic cardiomyopathy, the arrhythmogenic substrate often is located closer to the epicardial surface, beyond the reach of endocardial radiofrequency ablation.^2^ Access to the epicardial surface of the heart may be achieved through a subxiphoid puncture, either directly by puncture of the small fluid-filled space between the two layers of the pericardial sac^3^ or after insufflation of carbon dioxide into the pericardial space through an intentional perforation from a coronary sinus branch.^4^ We describe a dramatic complication that appeared several weeks after a redo epicardial ablation procedure. The data underlying this article are available in the article.

## Summary figure

**Table:** 

*Since many years*	Dilated cardiomyopathy, Implanted dual-chamber implantable cardioverter defibrillator (ICD)
*Minus 2.5 years*	Endoepicardial catheter ablation of ventricular tachycardias
*Minus 2 months*	Relapses of ventricular tachycardias documented at exercise electrocardiogram
*Time 0*	Redo epicardial catheter ablation
*4 days*	Discharge from hospital (uneventful immediate post-ablation period)
*42 days*	First medical contact for lower back and abdominal pain. Discharged with painkillers
*47 days*	Hospitalized at local hospital due to increasing pain
*48 days*	CT scan performed
*49 days*	Transferred to University Hospital
*50 days*	Emergency abdominal surgery after clinical cardiac arrest
*63 days*	Discharged from hospital after recovery
*93 days*	Clinical evaluation and ICD control at outpatient clinic

## Case report

Our patient is a 67-year-old male with dilated cardiomyopathy (DCMP) for 39 years. He is a former athlete and professional trainer, who works part-time teaching athletics. Aside from a cerebral embolic episode 23 years ago, without any neurological residuals, he has been healthy, exercised regularly, and particularly with no previous gastrointestinal symptoms. His DCMP initiated with VTs and slightly reduced left ventricular function that has deteriorated gradually. Treatment has been tried with antiarrhythmic and heart failure drugs, and a dual-chamber implantable cardioverter defibrillator (ICD) implanted for 14 years. He experienced frequent VTs including VT storm despite chronic treatment with amiodarone. We performed an endocardial catheter ablation procedure 2.5 years ago without clinical success, while a subsequent epicardial ablation targeting inferolateral late potentials rendered him arrhythmia-free for 2 years.

**Figure 1:**
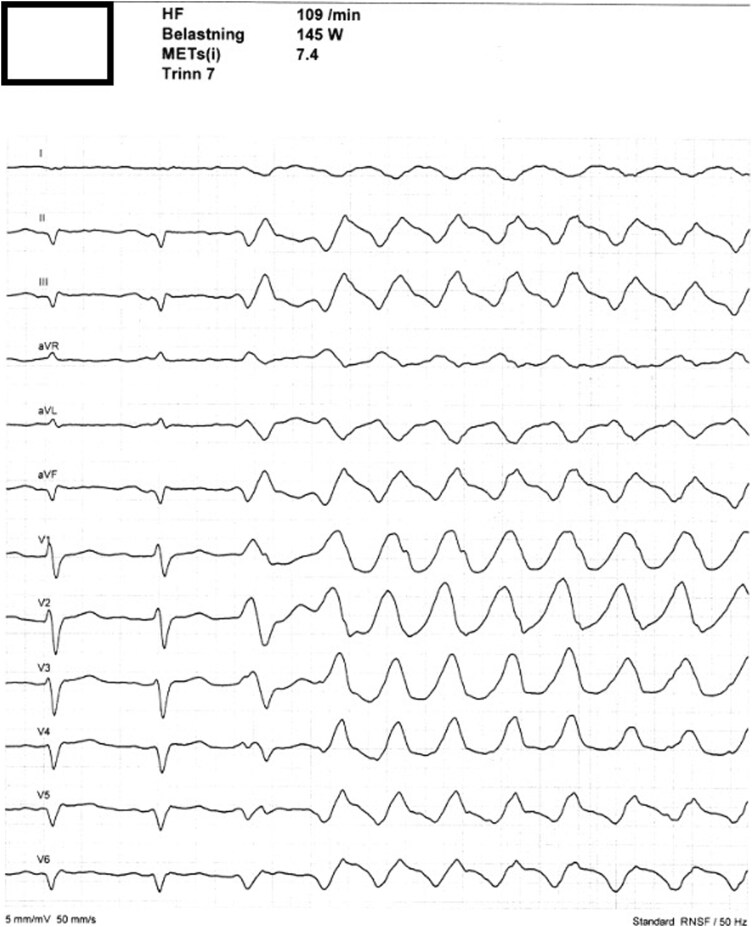
Twelve-lead electrocardiogram during moderate bicycle exercise test (145 W, 7.4 METs) showing sinus tachycardia followed by ventricular tachycardia at a heart rate of 185.

He was reluctant to stop the treatment with amiodaron but accepted a dose reduction from 200 to 100 mg daily. However, after 2 years, he started to complain about spells of rapid heart rate during training, like previous VT episodes. Implantable cardioverter defibrillator recordings showed non-sustained VT (cycle length 320 ms), and exercise electrocardiogram (ECG) demonstrated VT with right bundle branch block morphology and right inferior axis (*Figure 1*). A redo ablation was requested. At admission, he had an echocardiographic ejection fraction of 25–30%. His medication included amiodarone, flecainide, bisoprolol, sacubitril–valsartan, dapagliflozin, and apixaban *Figure 1*.

**Figure 2:**
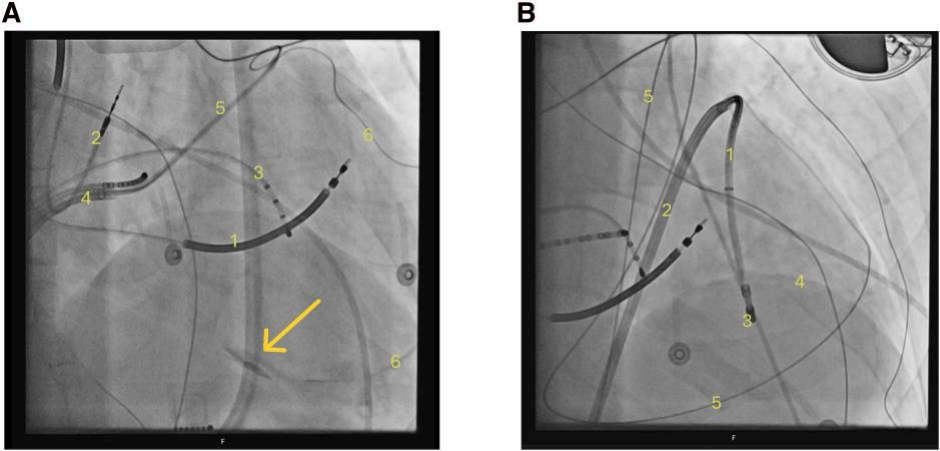
(*A*) Right anterior oblique view of the heart with implantable cardioverter defibrillator electrodes in the right ventricle (1) and right atrium (2), a penta-polar pacing catheter in the right ventricle (3), and steerable sheath in the coronary sinus ostium (4). A JR4 guiding catheter (5) with percutaneous coronary intervention (PCI)-wire and a microcatheter (6) was used to cannulate a coronary sinus side branch and intentionally perforate to the epicardial space, verified by contrast injection (arrow). CO_2_ gas was injected through the microcatheter. The gas is visible by fluoroscopy and creates space for transcutaneous puncture from the subxiphoid position with diminished risk of touching the cardiac surface with the needle. (*B*) Left anterior oblique view of the heart with ablation catheter (1) through the Agilis epi steerable sheath (2) into the epicardial space along the posterolateral wall of the left ventricle, with tip electrode in ablated area (3). The dome of the left diaphragm (4) is visible beneath the heart shadow. An additional guidewire (5) in the epicardial space secures access in case of dislocation and outlines the epicardial space.

**Figure 3:**
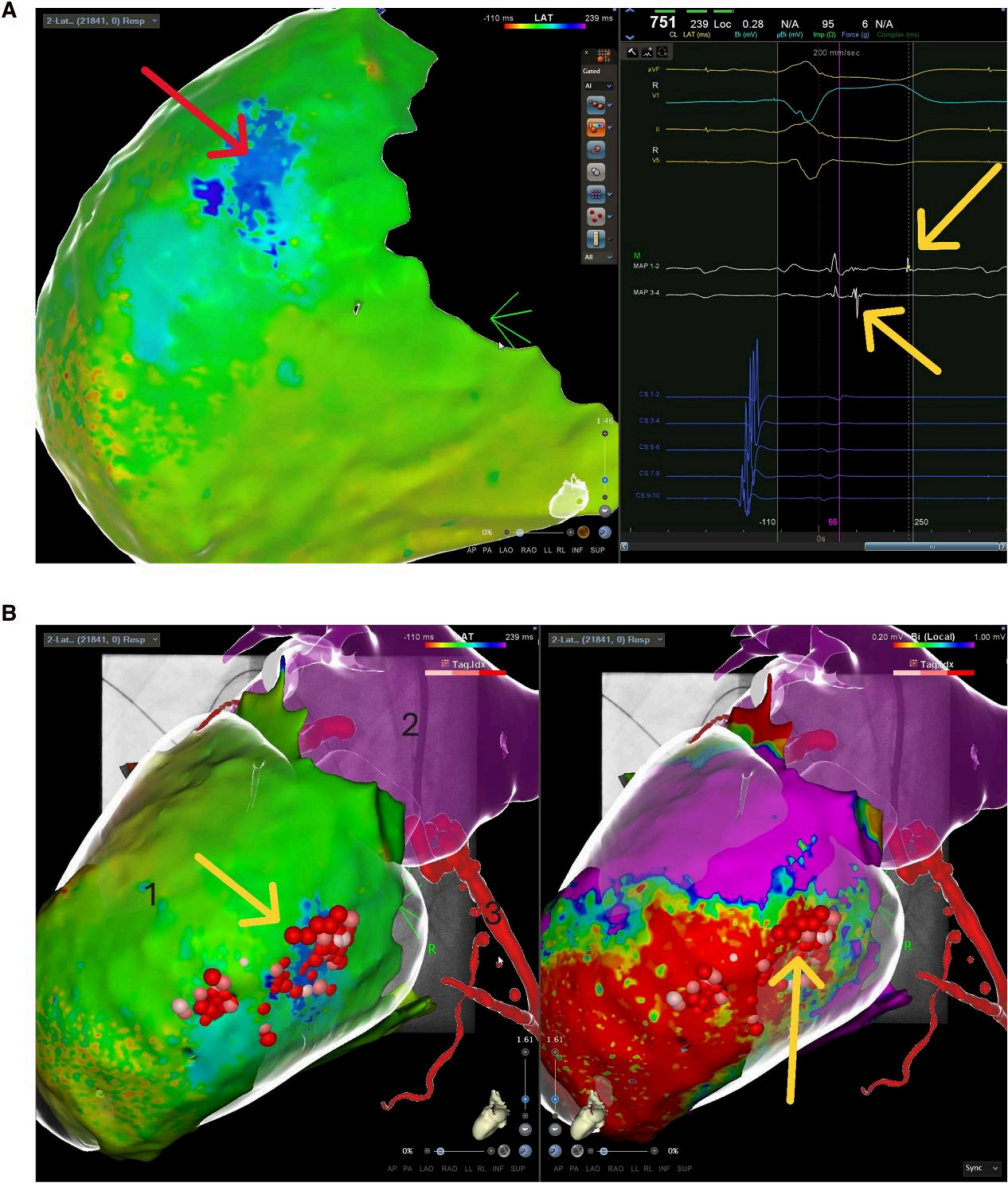
(*A*) Activation map of the epicardial surface of the left ventricle (posterior view) with blue colour (red arrow) indicating the region with the most delayed potentials. This is also illustrated with distinct, sharp signals from the distal and proximal bipolar electrodes of the ablation catheter (Map 1–2 and Map 3–4, yellow arrows) occurring after the surface QRS signal. (*B*) Synchronous activation map (left) and voltage map (right) in a left posterior oblique view, including the left ventricle (1) and atrium (2) merged with a computed tomography angiography showing the epicardial surface and the left coronary artery (3). The voltage map displays normal voltage in violet tint, low-voltage scar/fibrous tissue in red, and the border zone in blue, green, and yellow. The red and pink dots (yellow arrows) represent tags at the locations where we delivered radiofrequency energy to eliminate the ventricular tachycardia.

We performed a new ablation procedure, with anterior epicardial access through a subxiphoid puncture following the intentional perforation of a coronary sinus branch and the insufflation of 150 mL carbon dioxide (*Figure 2A* and *B*). Three-dimensional (3D) mapping was achieved using an Agilis epi steerable sheath (Abbott Cardiovascular), the Carto III-system with version 7 software (Johnson & Johnson MedTech), and a Decanav mapping catheter [Biosense Webster (BW)]. The epicardial 3D map was integrated with a computed tomography (CT) angiography scan to visualize the cardiac chambers and coronary arteries. No VT was inducible at the initiation of the procedure, but distinct delayed potentials were found in a limited area on the inferior basal and posterolateral surface of the left ventricle (*Figure 3A*). A large area apically and inferolaterally had only low voltage and no late potentials. The entire epicardial space was accessible, with no adhesions from the previous epicardial procedure.

Ablation of delayed potentials was performed with the aim of substrate modification, with Thermocool ST SF catheter (BW), 30–40 W for a total duration of 2345 s. At the end of the procedure, triamcinolone 160 mg was instilled in the pericardial space prior to withdrawal of the epicardial sheath, according to our routine.

The immediate post-procedural period was uneventful, and the patient was discharged on the fourth postoperative day. He experienced some gastrointestinal disturbances during the first week, likely due to 0.5 mg colchicine taken twice daily for 1 week as prophylaxis against pericarditis. Thereafter, he recovered to his habitual state.

**Figure 4:**
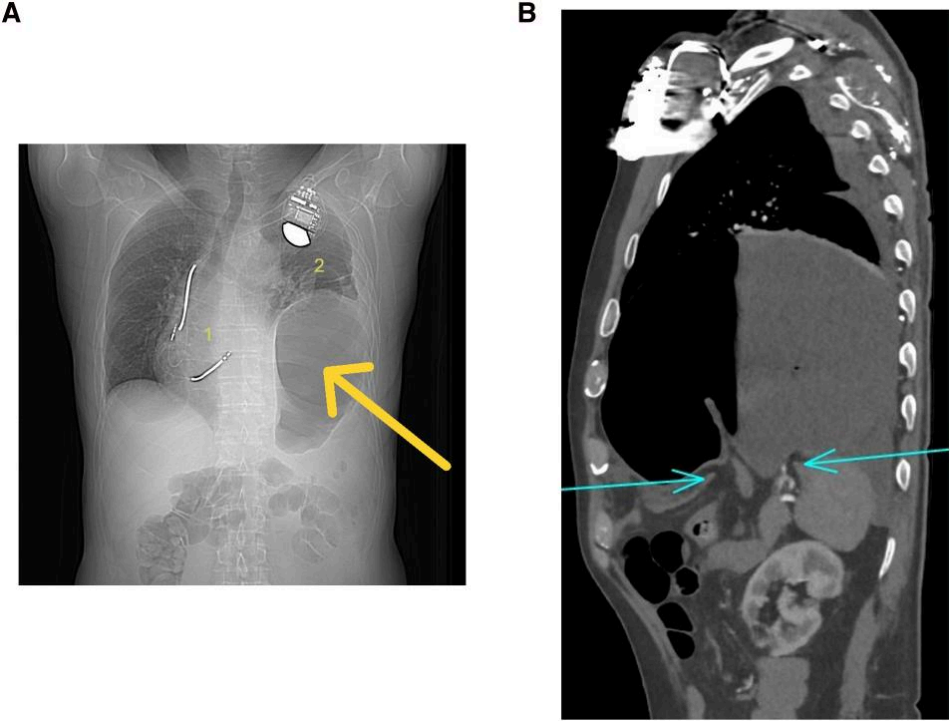
(*A*) Anteroposterior computed tomography scout view with the herniation of abdominal organs into the left thoracic cavity (yellow arrow), displacing the heart (1) towards right and the left lung (2) superiorly. (*B*) Sagittal computed tomography view with blue arrows marking the hernial opening, and abdominal organs extending into the left thoracic cavity.

Six weeks after the ablation procedure, the patient experienced acute lower back and abdominal pain. He visited a primary care physician and was dismissed with painkillers only. However, the symptoms persisted and worsened by eating and drinking. After 5 more days, he was hospitalized at his local hospital. Upon presentation, the patient reported severe post-prandial epigastric and left hypochondrium pain, radiating to the interscapular area. Blood pressure was 116/84 mmHg and heart rate was 70 b.p.m. C-reactive protein was elevated to 82 mg/L (reference interval 0–5 mg/L), troponin I 1224 mg/L (ref. <30 ng/L), and NT-proBNP 3279 ng/L (ref. <376 ng/L). The initial differential diagnoses included aortic dissection, oesophageal fistula, or other post-procedural complications. Chest X-ray showed atelectasis in the lower part of the left lung, and an elevated contour from the stomach initially interpreted as a new hiatic hernia. Computed tomography angiography of the aorta the following day demonstrated a left-sided diaphragmatic hernia that had not been present on pre-ablation chest X-rays or cardiac CT angiographies. The entire stomach, along with a small portion of the splenic flexure of the colon, had herniated through ∼6 cm defect in the diaphragm, resulting in displacement of the mediastinum and lungs (*Figure 4A* and *B*). There was no evidence of twisting of the distal oesophagus or proximal duodenum, and no gastric wall dilatation or signs of perforation. However, the stomach was distended with air–fluid levels. An upper endoscopy showed gastric retention without mucosal injury, and a nasogastric tube was placed for decompression of the stomach.

The following day, the patient was transferred to the Department of Gastrointestinal Surgery at our hospital. The on-call surgeon found no need for emergency surgery because the stomach was decompressed, and the complex cardiological situation required specialized anaesthesiologic competence. A new echocardiography and upper endoscopy were performed, and laparoscopic surgery was planned. However, within 24 h from the transferal, the patient suddenly deteriorated, experiencing malaise, hypotension, and cardiac arrest. Resuscitation was immediately initiated and return of spontaneous circulation was achieved after 7 min. Subsequently, emergency surgery was performed on vital indication, with laparotomy that revealed free blood in the peritoneal space from splenic haemorrhage. A splenectomy was performed; the stomach was apparently healthy within the abdomen. There was a 5–7 cm long linear defect with relatively raw edges in the diaphragm, transversally from the middle of the left crus. The defect was repaired by direct suture with non-resorbable material. Due to postoperative fever of 39°C, elevated white blood cell count 16.1 × 10^9^/L (reference 3.5–10 × 10^9^/L), elevated C-reactive protein 224 mg/L (reference 0–4 mg/L), and the fact that the patient had been critically ill with cardiopulmonary resuscitation, he was empirically treated with piperacillin/tazobactam (Fresenius Kabi) for 5 days while waiting for bacterial tests grown from both blood and tracheal secretions. No pathogenic bacteria were detected. He was extubated the first postoperative day, gradually mobilized, and recovered remarkably well except for some posttraumatic stress response that was taken care of by the Department for Psychosomatic Care. Before discharge, the patient was vaccinated against pneumococci and meningococci type B according to the local routine after splenectomy.

One month after the surgery, the patient came to a routine follow-up at the cardiological outpatient clinic. He was exercising regularly and able to walk for 3–5 km. Interrogation of the ICD revealed no VTs since the ablation. His heart failure was well compensated with sacubitril/valsartan, beta-blocker, and a sodium–glucose-linked transporter inhibitor.

## Discussion

We report a rare complication that occurred several weeks after an epicardial ablation procedure, which we had not previously heard of. However, at the same time as our patient was operated, Ghirardelli *et al*.^5^ published a similar case report from Italy. In their case, the patient had undergone both endocardial and epicardial ablation some years earlier, but the last procedure was exclusively endocardial. The ablation target inferiorly and inferolaterally is like our case, in DCMP related to previous myocarditis. The rupture in this case occurred 2 days post-ablation during hospitalization, while our case appeared 6 weeks after discharge. The initial symptom reported from Italy was multiple episodes of vomiting and coughing, and then haematemesis. Ghirardelli does not mention the use of CO_2_ insufflation; at that time (2017), epicardial access was commonly obtained by direct puncture without CO_2_. The location of the diaphragmatic hernia in our patient is anatomically closely related to the ablation lesions on the inferior basal and posterolateral surface of the left ventricle (*Figure 3A* and *B*). We have reviewed the pre-ablation CT angiography and could not find any previous diaphragmic pathology. We hypothesize that the hernia resulted from collateral heating during the second epicardial ablation procedure. This may have occurred despite our consistent practice directing the ablation vector towards the myocardium to maximize the effect of ablation and avoid collateral damage. Previous endocardial and epicardial ablations in the same area also may have contributed. Notably, both our case and the case from Italy had endocardial and epicardial ablation performed a couple of years previously. The energy settings of 30–40 W were similar in our previous epicardial procedure, while we sometimes deliver up to 50 W in endocardial ablation. CO_2_ insufflation in the pericardial space creates an elevated pressure inside the pericardial sack for a limited period while puncture is performed. The pressure is normalized as soon as access to the pericardial space is achieved and CO_2_ diffuses away. In our opinion, this hardly represents any mechanical injury to the diaphragm. The puncture site was anteriorly and superiorly behind the sternum, which is far away from the dome of the diaphragm and probably not related to muscular weakening. Inadvertent direct mechanical injury to the diaphragm from the ablation catheter in the epicardial space cannot be entirely excluded, although a 6-week delay makes this mechanism unlikely. In the case report by Ghirardelli, the rupture occurred only 2 days after the procedure, but the last procedure had been endocardial only.

When our patient was hospitalized in the local hospital, a chest X-ray and CT scan of the whole aorta, including both the thoracic and abdominal cavities, were requested due to a wide range of differential diagnoses including aortic dissection, oesophageal pathology, and post-procedural complications. The demonstration of a diaphragmatic hernia was unexpected. An abdominal ultrasonographic examination could potentially have given valuable insight but was not performed.

There was an unfortunate 48 h delay from the CT angiography being performed until surgery was completed. The patient had a life-threatening cardiac arrest necessitating immediate open surgery. Several factors may have contributed, like hospitalization during Easter Holidays, transferal from the local hospital to the University Hospital, and the fact that the Electrophysiological Department and Department of Gastrointestinal Surgery are located at different campuses within the University Hospital. The haemorrhage of the spleen probably was caused by the sliding of abdominal organs back and forth through the diaphragmatic hernia and likely caused the final deterioration with haemodynamic collapse. It is possible that this complication might have been avoided if the patient had been operated on earlier during the hospital stay. Our case report highlights the importance of immediate surgery of acute diaphragmatic hernias at a hospital with the ability to perform combined thoracoabdominal surgery and experience with circulatory support.

Current literature report complication rates of 1–14% related to epicardial catheter ablation^6^ with right ventricular puncture, acute haemopericardium, and acute tamponade as the most common. Less frequent complications include coronary artery injuries, phrenic nerve paralysis, and accidental puncture of abdominal organs with haematomas. Electrophysiologists are aware of the possibility of collateral damage to the oesophagus when treating patients with atrial fibrillation–related arrhythmia, using radiofrequency energy on the posterior left atrial wall. Atrio-oesophageal fistula is a rare complication with a high mortality rate, and typical clinical presentation 2–4 weeks after the ablation procedure.^7^ The present case report also is notable for the delayed clinical presentation, occurring after the patient had left the hospital and recovered well. Procedure-related complications may appear several weeks after the procedure was performed, and it is important to diagnose these rare cases and treat them properly.

## Conclusion

We describe a rare, but life-threatening complication to epicardial catheter ablation. Iatrogenic diaphragmatic perforation may occur several weeks after the ablation procedure, and the first healthcare providers may have limited knowledge about catheter ablation and potential complications. Therefore, the patients should be instructed on how to respond if severe, unexpected symptoms occur after the procedure.

## Data Availability

Data available on request.

## Lead author biography

Ole-Gunnar Anfinsen is a senior consultant cardiologist and electrophysiologist at Oslo University Hospital Rikshospitalet. His main interest is catheter ablation of various cardiac arrhythmias including atrial fibrillation and ventricular tachycardias in patients with structural heart disease.

## Notes

**Consent:** The patient has consented to the publication of this case report.

## References

[1] Sapp JL, Tang ASL, Parkash R, Stevenson WG, Healey JS, Gula LJ, et al Catheter ablation or antiarrhythmic drugs for ventricular tachycardia. N Engl J Med 2024;392:737–747. 39555820 10.1056/NEJMoa2409501

[2] Della Bella P, Brugada J, Zeppenfeld K, Merino J, Neuzil P, Maury P, et al Epicardial ablation for ventricular tachycardia: a European multicenter study. Circ Arrhythmia Electrophysiol 2011;4:653–659. 10.1161/CIRCEP.111.962217 21841191

[3] Sosa E, Scanavacca M, D’Avila A, Pilleggi F. A new technique to perform epicardial mapping in the electrophysiology laboratory. J Cardiovasc Electrophysiol 1996;7:531–536. 8743758 10.1111/j.1540-8167.1996.tb00559.x

[4] Juliá J, Bokhari F, Uuetoa H, Derejko P, Traykov VB, Gwizdala A, et al A new era in epicardial access for the ablation of ventricular arrhythmias: the Epi-CO2 registry. JACC Clin Electrophysiol 2021;7:85–96. 33478716 10.1016/j.jacep.2020.07.027

[5] Ghirardelli L, Genova L, Angelo GD, Bisceglia C, Carlucci M. A case report of a strangulated diaphragmatic laceration : an uncommon late complication of cardiac ablation. Report 2025;8:48. 10.3390/reports8020048 PMC12196956 40710839

[6] Bisceglia C, Limite LR, Baratto F, D’Angelo G, Cireddu M, Della Bella P. Road-map to epicardial approach for catheter ablation of ventricular tachycardia in structural heart disease: results from a 10-year tertiary-center experience. Circ Arrhythmia Electrophysiol 2024;17:e012181. 10.1161/CIRCEP.123.012181 38836351

[7] Schuring CA, Mountjoy LJ, Priaulx AB, Schneider RJ, Smith HL, Wall GC, et al Atrio-esophageal fistula: a case series and literature review. Am J Case Rep 2017;18:847–854. 28761039 10.12659/AJCR.903966 PMC5551930

